# Supplementary material for: Intra- and Interspecies RNA-Seq Based Variants in the Lactation Process of Ruminants
Source: Animals (Basel). 2022 Dec 19;12(24):3592. doi: 10.3390/ani12243592 (PMC9774614; doi:10.3390/ani12243592)
Supplement: Supplementary file 1 [file animals-12-03592-s001.zip › Supplementary Table S1.pdf]

Investigation of intra- and interspecies transcriptomic variants in the cow and sheep lactation process

Mohammad Farhadian*[1], Seyed Abbas Rafat[1],

1-Department of Animal Science, Faculty of Agriculture, University of Tabriz, Tabriz, Iran

***Corresponding author:**

Mohammad Farhadian, Department of Animal Science, Faculty of Agriculture, University of Tabriz, Tabriz, Iran.

Tel: +98 9149765639

Email: Mohammad.farhadian@tabrizu.ac.ir

Supplementary Table S1. Detailed information of the datasets

Accession ID	Species	Stage	Run Accession	Read Count	mapping rate
SRP125676	Bos Taurus (Jersy)	BP	SRR6324365	48124770	95.7%
			SRR6324366	45607023	92.1%
			SRR6324367	45302553	92.7%
		P	SRR6324368	46258686	90.1%
			SRR6324369	46617564	88.2%
		AP	SRR6324370	58200765	91.0%
			SRR6324371	68414217	91.5%
			SRR6324372	38824961	90.0%
SRP125676	Bos Taurus (Kashmiri)	BP	SRR6324373	41231387	85.7%
			SRR6324374	40905980	86.7%
			SRR6324375	52285576	87.7%
		P	SRR6324376	36943668	90.0%
			SRR6324377	34212918	85.4%
			SRR6324378	38487367	90.4%
		AP	SRR6324379	46049214	93.1%
			SRR6324379	43860412	91.1%
			SRR6324381	60444037	90.9%

Accession ID	Species	Stage	Run Accession	Read Count	mapping rate
SRP065967	Ovis aries (Assaf)	BP	SRR2932535	35043772	95.6%
			SRR2932536	33159147	97.15%
			SRR2932537	31081902	96.58%
			SRR2932538	41322109	96.18%
		P	SRR2932543	40313876	96.60%
			SRR2932544	42580059	96.60%
			SRR2932545	29793742	95.40%
			SRR2932546	34921005	96.23%
		AP	SRR2932551	41394140	96.55%
			SRR2932552	38933690	95.99%
			SRR2932553	36187490	96.52%
			SRR2932557	37213653	96.61%
			SRR2932558	29991596	96.40%
			SRR2932559	40861735	96.53%
			SRR2932560	35432575	96.39%
SRP065967	Ovis aries (Churra)	BP	SRR2932539	30130200	96.90%
			SRR2932540	28025028	94.9%
			SRR2932541	36170492	92.3%
			SRR2932542	23297744	96.23%
			SRR2932547	29180331	94.61%
			SRR2932548	45690280	90.1%
		P	SRR2932549	45967224	88.0%

			SRR2932550	42476719	96.84%
		AP	SRR2932554	35170966	96.63%
			SRR2932555	39951447	90.3%
			SRR2932556	33519563	89.5%
			SRR2932561	31562685	97.08%
			SRR2932562	42943369	94.3%
			SRR2932563	35600309	90.9%
			SRR2932564	43217874	89.9%

BP: before peak, P: peak, AP: after peak
