# Supplementary material for: Intra- and Interspecies RNA-Seq Based Variants in the Lactation Process of Ruminants
Source: Animals (Basel). 2022 Dec 19;12(24):3592. doi: 10.3390/ani12243592 (PMC9774614; doi:10.3390/ani12243592)
Supplement: Supplementary file 1 [file animals-12-03592-s001.zip › Supplementary Table S2.pdf]

Investigation of intra- and interspecies transcriptomic variants in the cow and sheep lactation process

Mohammad Farhadian*[1], Seyed Abbas Rafat[1],

1-Department of Animal Science, Faculty of Agriculture, University of Tabriz, Tabriz, Iran

***Corresponding author:**

Mohammad Farhadian, Department of Animal Science, Faculty of Agriculture, University of Tabriz, Tabriz, Iran.

Tel: +98 9149765639

Email: Mohammad.farhadian@tabrizu.ac.ir

Supplementary Table S2. Annotated missense variants in major milk protein genes

Gene	Breed/stage	Variant	Deleterious	Amino acids	Codons
PAEP	BP/P/AP-Assaf/ Churra	rs430610497	NO	H/Y	Cac/Tac
PAEP	BP/P/AP -Kashmiri	rs109625649	NO	A/V	gCc/gTc
PAEP	BP-Jersy	rs109625649	NO	A/V	gCc/gTc
LALBA	BP/P-Assaf/ Churra	rs403176291	NO	A/V	gCg/gTg
LALBA	BP/P/AP-Kashmiri	rs465119286	NO	I/V	Ata/Gta
LALBA	AP-Kashmiri	rs722550244	NO	R/Q	cGg/cAg
CSN2	BP/AP-Jersy	rs43703013	NO	R/S	agG/agC
CSN2	BP/AP-Jersy	rs43703011	NO	H/P	cAt/cCt
CSN2	BP-Jersy	rs109299401	NO	M/L	Atg/Ctg
CSN2	BP/P/AP-Kashmiri	rs43703013	NO	R/S	agG/agC
CSN2	BP/P/AP-Kashmiri	rs43703011	NO	H/P	cAt/cCt
CSN3	BP/P/AP-Jersy	rs43703016	YES	A/D	gCt/gAt
CSN3	BP/P/AP-Jersy	rs43703015	NO	I/T	aTc/aCc
CSN3	BP/P/AP-Kashmiri	rs43703016	YES	A/D	gCt/gAt
CSN3	BP/P/AP-Kashmiri	rs43703015	NO	I/T	aTc/aCc
CSN3	AP-Kashmiri	rs450402006	NO	T/I	aCc/aTc
CSN1S1	BP/P/AP-Kashmiri	rs43703010	NO	E/G	gAa/gGa
CSN1S1	AP-Jersy	rs43703010	NO	E/G	gAa/gGa
CSN1S2	AP-Jersy	rs465436451	NO	T/A	Act/Gct
CSN1S2	AP-Kashmiri	rs476152522	NO	V/F	Gtt/Ttt

BP: before peak, P: peak, AP: after peak
