# Supplementary material for: Intra- and Interspecies RNA-Seq Based Variants in the Lactation Process of Ruminants
Source: Animals (Basel). 2022 Dec 19;12(24):3592. doi: 10.3390/ani12243592 (PMC9774614; doi:10.3390/ani12243592)
Supplement: Supplementary file 1 [file animals-12-03592-s001.zip › Supplementary Table S3.pdf]

Investigation of intra- and interspecies transcriptomic variants in the cow and sheep lactation process

Mohammad Farhadian*[1], Seyed Abbas Rafat[1],

1-Department of Animal Science, Faculty of Agriculture, University of Tabriz, Tabriz, Iran

***Corresponding author:**

Mohammad Farhadian, Department of Animal Science, Faculty of Agriculture, University of Tabriz, Tabriz, Iran.

Tel: +98 9149765639

Email: Mohammad.farhadian@tabrizu.ac.ir

Supplementary Table S3. Annotated missense variants in major milk fat genes

Gene	Breed/stage	Variant	Deleterious	Amino_acids	Codons
ACACA	BP-Assaf	rs1091823970	NO	A/T	Gca/Aca
ACSL1	BP/P-Assaf	rs398916864	NO	I/V	Att/Gtt
ACSL1	BP/P/AP-Churra	rs398916864	NO	I/V	Att/Gtt
ACSL1	BP/AP-Kashmiri	rs445326585	NO	I/V	Att/Gtt
LPL	BP/P-Assaf	rs160168269	NO	A/V	gCc/gTc
LPL	BP/P/AP-churra	rs160168269	NO	A/V	gCc/gTc
ACSS2	BP/P/AP-Assaf	rs410061432	NO	P/L	cCg/cTg
ACSS2	BP/P/AP-churra	rs410061432	NO	P/L	cCg/cTg
ACSS2	BP/P/AP-Kashmiri	rs718091805	NO	P/Q	cCa/cAa
ACSS2	BP/P/AP-Kashmiri	rs520341778	NO	R/Q	cGa/cAa
XDH	BP/P-Assaf	rs429850918	YES	R/W	Cgg/Tgg
XDH	BP/P/AP-Assaf	rs428221119	YES	L/F	ttG/ttC
XDH	BP/P/AP-Assaf	rs418262376	NO	S/N	aGt/aAt
XDH	BP/P/AP-churra	rs422371829	NO	A/T	Gca/Aca
XDH	P/AP-churra	rs420294440	NO	T/S	aCt/aGt
XDH	BP-churra	rs401680847	NO	R/Q	cGg/cAg
XDH	BP/P-Jersy	rs42890834	NO	R/K	aGg/aAg
XDH	BP-Jersy	rs110613653	NO	E/Q	Gag/Cag
XDH	BP-Kashmiri	rs720370046	NO	M/T	aTg/aCg
XDH	P/AP/BP-Kashmiri	rs714668250	NO	Q/K	Cag/Aag
XDH	BP/P/AP-Kashmiri	rs42890834	NO	R/K	aGg/aAg
XDH	BP/P/AP-Kashmiri	rs110613653	NO	E/Q	Gag/Cag
XDH	P/AP-Kashmiri	rs720370046	NO	M/T	aTg/aCg
GPAM	BP/P-Assaf	rs401931673	NO	V/I	Gtc/Atc
GPAM	BP/P-Assaf	rs161498056	NO	N/S	aAc/aGc
GPAM	P-Assaf	rs162244594	NO	K/N	aaG/aaC
GPAM	BP/AP-churra	rs401931673	NO	V/I	Gtc/Atc
GPAM	BP-churra	rs162244594	NO	K/N	aaG/aaC
GPAM	BP/P/AP-churra	rs161498056	NO	N/S	aAc/aGc
GPAM	BP-Jersy	rs109886010	YES	R/W	Cgg/Tgg
GPAM	BP-Kashmiri	rs518964599	NO	R/K	aGg/aAg
FASN	BP/P/AP-Assaf	rs419446704	NO	Q/E	Cag/Gag
FASN	BP/P-Jersy	rs720932463	YES	S/R	agC/agG
FASN	BP/P-Jersy	rs715140536	NO	A/T	Gcc/Acc
FASN	BP/P-Jersy	rs526101337	NO	E/K	Gag/Aag
FASN	BP/P-Jersy	rs523890615	NO	S/G	Agc/Ggc
FASN	BP/P-Jersy	rs516607144	YES	L/F	Ctc/Ttc
FASN	BP/P-Jersy	rs41919993	NO	Y/H	Tac/Cac
FASN	BP/P-Jersy	rs41919985	NO	T/A	Acc/Gcc
FASN	BP/P-Jersy	rs209734560	YES	R/W	Cgg/Tgg
FASN	BP/P-Jersy	rs209227647	NO	R/H	cGc/cAc
FASN	BP/P-Jersy	rs208645216	NO	A/T	Gca/Aca
FASN	P-Jersy	rs109149276	NO	L/I	Ctt/Att
FASN	BP/P/AP-Kashmiri	rs718745442	NO	R/G	Aga/Gga

FASN	BP/P/AP-Kashmiri	rs715140536	NO	A/T	Gcc/Acc
FASN	BP/P/AP-Kashmiri	rs526101337	NO	E/K	Gag/Aag
FASN	BP/P/AP-Kashmiri	rs523890615	NO	S/G	Agc/Ggc
FASN	BP/P/AP-Kashmiri	rs516607144	YES	L/F	Ctc/Ttc
FASN	BP/P/AP-Kashmiri	rs481622676	NO	A/G	gCt/gGt
FASN	BP/P/AP-Kashmiri	rs41919993	NO	Y/H	Tac/Cac
FASN	BP/P/AP-Kashmiri	rs41919985	NO	T/A	Acc/Gcc
FASN	BP/P/AP-Kashmiri	rs209227647	NO	R/H	cGc/cAc
FASN	BP/P/AP-Kashmiri	rs109149276	NO	L/I	Ctt/Att
FASN	AP-Kashmiri	rs716641373	NO	V/I	Gtc/Atc
FASN	AP-Kashmiri	rs462083425	NO	R/H	cGc/cAc
FASN	AP-Kashmiri	rs450506819	NO	I/S	aTc/aGc
FASN	AP-Kashmiri	rs211379310	NO	A/T	Gcc/Acc
VLDLR	BP-Assaf	rs406299573	NO	A/T	Gcc/Acc
PLIN2	BP/P-churra	rs160139616	NO	K/R	aAa/aGa
PLIN2	BP/P-Assaf	rs160139616	NO	K/R	aAa/aGa
PLIN2	BP/P-Jersy	rs42211560	NO	A/V	gCt/gTt
PLIN2	BP-Jersy	rs211616654	NO	M/T	aTg/aCg
PLIN2	BP/P-Jersy	rs110584382	NO	R/Q	cGg/cAg
PLIN2	BP/P/AP-Kashmiri	rs42211560	NO	A/V	gCt/gTt
PLIN2	BP/P/AP-Kashmiri	rs380664726	YES	R/W	Cgg/Tgg
PLIN2	BP/P/AP-Kashmiri	rs211616654	NO	M/T	aTg/aCg
PLIN2	BP/P/AP-Kashmiri	rs210208890	NO	R/T	aGa/aCa
PLIN2	AP-Kashmiri	rs525585406	YES	K/E	Aag/Gag
PLIN2	AP-Kashmiri	rs523116647	NO	R/Q	cGa/cAa
PLIN2	AP-Kashmiri	rs516757589	NO	K/R	aAg/aGg
BTN1A1	BP-churra	rs403441604	NO	E/K	Gag/Aag
BTN1A1	BP/P-Jersy	rs441004576	NO	V/I	Gtc/Atc
BTN1A1	BP/P-Jersy	rs43708439	NO	D/E	gaT/gaA
BTN1A1	BP/P-Jersy	rs211141026	NO	R/K	aGg/aAg
BTN1A1	BP/P-Jersy	rs209257029	NO	S/T	aGc/aCc
BTN1A1	BP/P-Jersy	rs208161697	NO	K/R	aAg/aGg
BTN1A1	BP/P-Jersy	rs109972486	NO	P/Q	cCg/cAg
BTN1A1	BP/P/AP-Kashmiri	rs109972486	NO	P/Q	cCg/cAg
BTN1A1	BP/P/AP-Kashmiri	rs799691258	NO	A/T	Gct/Act
BTN1A1	BP/P/AP-Kashmiri	rs519853022	NO	V/I	Gtc/Atc
BTN1A1	BP/P/AP-Kashmiri	rs43708439	NO	D/E	gaT/gaA
BTN1A1	BP/AP-Kashmiri	rs43706495	NO	R/H	cGt/cAt
BTN1A1	BP-Kashmiri	rs133113758	NO	A/T	Gcg/Acg

BP: before peak, P: peak, AP: after peak
